# Supplementary material for: Sphingolipid and Endocannabinoid Profiles in Adult Attention Deficit Hyperactivity Disorder
Source: Biomedicines. 2021 Sep 6;9(9):1173. doi: 10.3390/biomedicines9091173 (PMC8467584; doi:10.3390/biomedicines9091173)
Supplement: Supplementary file 1 [file biomedicines-09-01173-s001.zip › ADHD-SupplTables-SupplFigures_2021-09-03.pdf]

Supplementary Tables

Supplementary Table S1: Abbreviations

**Abbreviations**	
BMI	Body mass index
MD	Major depression
BP	Bipolar disorder
ECT	Electroconvulsive therapy
BDI	Beck Depression Inventory
YMRS	Young Mania Rating Scale
LC-ESI-MS/MS	Liquid chromatography-electrospray ionization-tandem mass spectrometry
LLOQ	Lower limit of quantification
ULOQ	Upper limit of quantification
QC	Quality control
IS	Internal standard
UHPLC	Ultrahigh performance liquid chromatography
m/z	Mass transition
Cer	Ceramide
GlcCer	Glucosylceramide
LacCer	Lactosylceramide
PCA	Principal component analysis
DA	Discriminant analysis
PLS	Partial least square analysis
ANOVA	Analysis of variance
CanDisc	Canonical discriminant score
CI	Confidence interval
CHAID	Chi-square automatic interaction detection

Abbreviations of analytes

Lipid name	Long name	Short name
AEA	Anandamide	AEA
OEA	Oleoylethanolamide	OEA
PEA	Palmitoylethanolamide	PEA
1AG / 2AG	1/2-Arachidonoylglycerol	1AG / 2AG
SPH d18:1/16:1	C16 Sphingosin-1-phosphate	S1P d16:1
SPH d18:1/18:1	C18 Sphingosin-1-phosphate	S1P d18:1
SPH d18:0/18:1	C18 Sphinganine-1-phosphate	S1P d18:0
Cer d18:1/16:0	C16 Ceramide	Cer16
Cer d18:1/18:0	C18 Ceramide	Cer18
Cer d18:1/20:0	C20 Ceramide	Cer20
Cer d18:1/22:0	C22 Ceramide	Cer22
Cer d18:1/24:0	C24 Ceramide	Cer24
Cer d18:1/24:1	C24:1 Ceramide	Cer24:1
GlcCer d18:1/16:0	16C Glucosylceramide	GluCer16
GlcCer d18:1/18:0	18C Glucosylceramide	GluCer18
GlcCer d18:1/24:1	C24:1 Glucosylceramide	GluCer24:1
LacCer d18:1/16:0	C16 Lactosylceramide	LacCer16
LacCer d18:1/18:0	C18 Lactosylceramide	LacCer18
LacCer d18:1/18:1	C18:1 Lactosylceramide	LacCer18
LacCer d18:1/24:0	C24 Lactosylceramide	LacCer24
LacCer d18:1/24:1	C24:1 Lactosylceramide	LacCer24:1

Supplementary Table S2: Drug treatments (Excel file)

Supplementary Figures and Legends

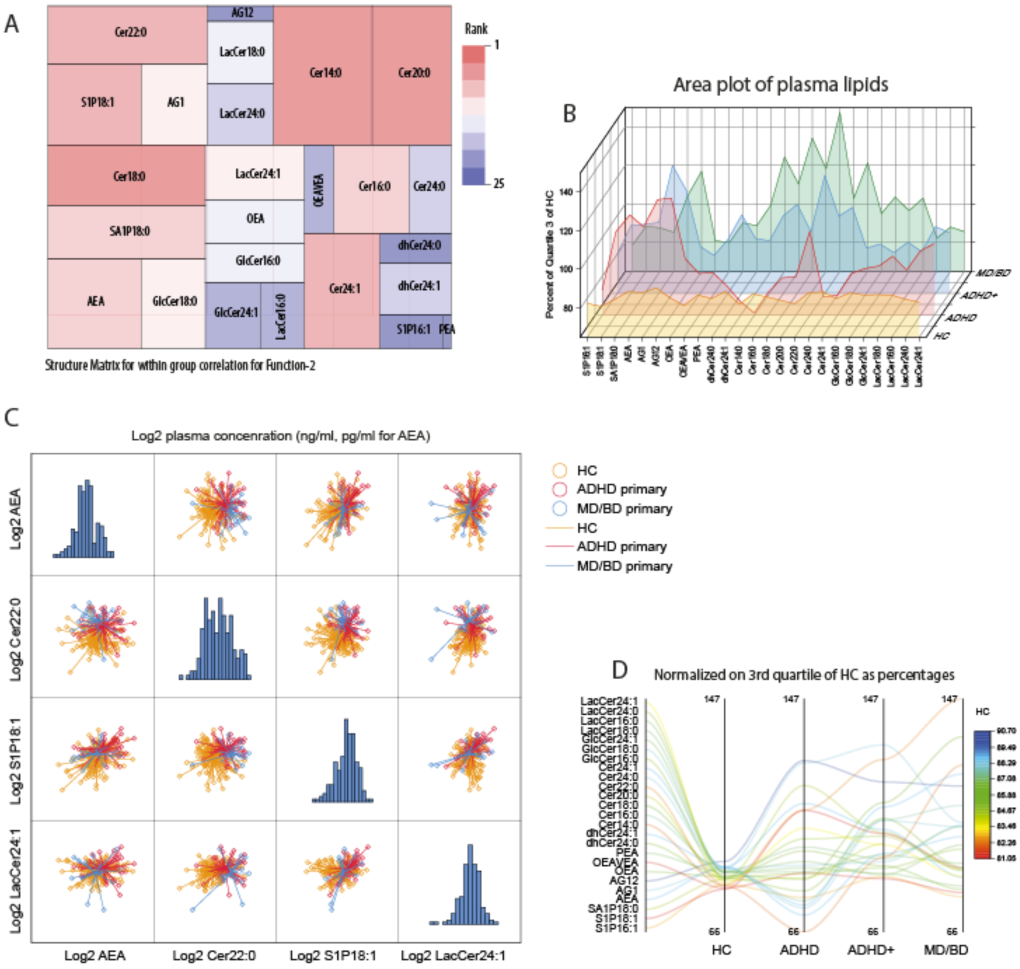

Supplementary Figure S1: Canonical discriminant analysis and lipid profiles

A: Tree map of the CanDisc structure matrix for function-2 (function-1 in Fig. 1B), which mainly discriminates between different patient groups. The sizes of the rectangles and the colors reveal the relative importance of the respective lipid for the discrimination of the groups, the sizes represent the within group correlations and the colors show the ranking. **B:** Waterfall plot of the lipid profiles. Lipid concentrations were normalized to the 75% quartile of the healthy controls, and they are presented as percentages. The respective area plot with error bars is shown in Fig. 1D. **C:** XY scatter plots with centroid spikes for candidate lipids. The plots show patients with attention deficits/hyperactivity disorder (ADHD), ADHD with a comorbidity of major depression (MD) or bipolar disorder (BD) (ADHD+), patients with MD/BD without ADHD, or healthy controls (HC). Each scatter is one subjects, the spikes originates from the groups' centroid. Samples sizes: n=49 ADHD or ADHD+, n=22 MD/BD, n=98 HC. The groups can be separated based on each two lipids, best separation is obtained from Cer22:0 by S1P d18:1, AEA by Cer22:0 and Cer22:0 by LacCer24:1. **D:** Line plots of normalized lipids. Concentrations were normalized as percentages of the 75% quartile of HC.

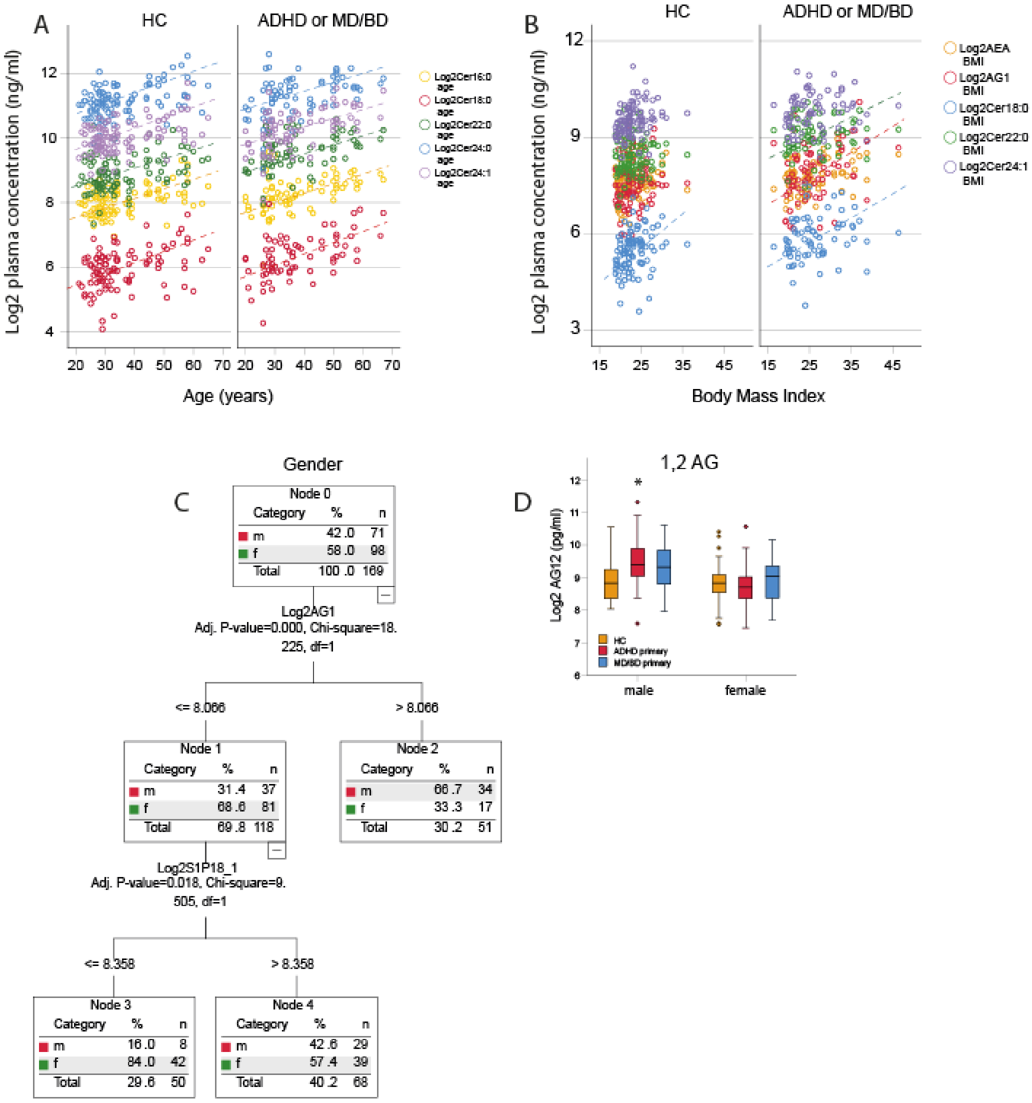

Supplementary Figure S2: Impact of gender, age and body may index

A: XY scatter plots show a linear increase of ceramide concentrations with age for all ceramides in healthy controls (HC) and in patients with attention deficit hyperactivity disorder (ADHD) without or with comorbidity, and in patients with major depression (MD) or bipolar disorder (BD). The dashed lines are the regression line. All slopes differed significantly from zero. Samples sizes: n=49 ADHD or ADHD+, n=22 MD/BD, n=98 HC. **B:** XY scatter plots show the associations of lipid concentrations with body mass index (BMI). Dashed lines show the regression lines for those lipids where the slopes differed significantly from zero. **C:** Decision tree of CHAID (Chi-square automatic interaction detection) analysis for gender. Male and female patients differ in 1-AG levels. **D:** Box plots of 1/2-AG grouped for sex. The box plots show the log2 plasma concentration of the sum of 1AG and 2AG. The box is the interquartile range (IQR), and the line is the median. The whiskers show the range, and the dots are outlier. Data were compared with MANOVA for "group" by "sex" and subsequently for group versus HC for each sex with adjustment of alpha according to Dunnett. *P<0.05. Sample sizes as in A.

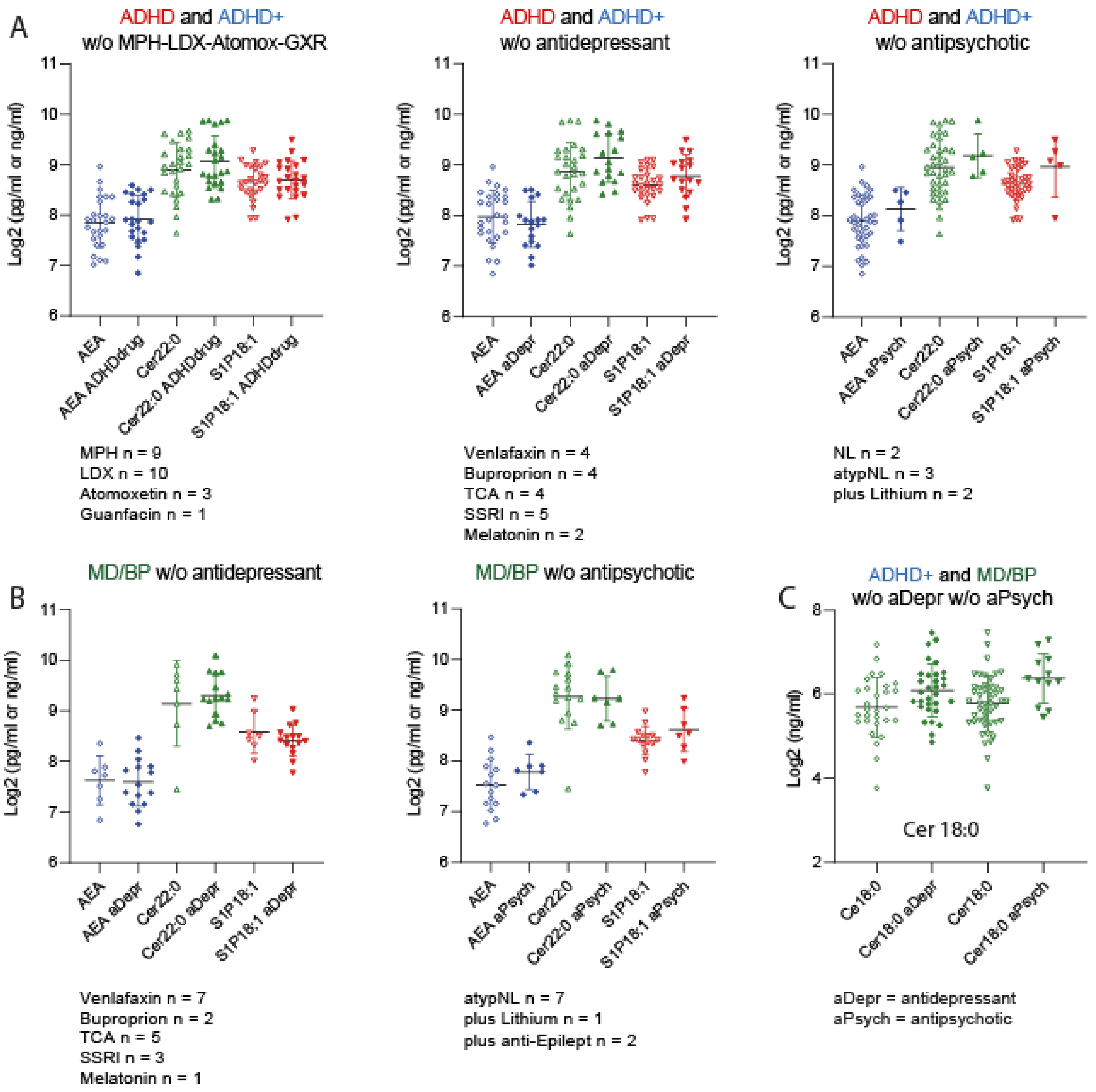

Supplementary Figure S3: Influence of drug treatments

A: Scatter plots of log2 plasma concentrations of the candidates lipids, anandamide (AEA in pg/ml), C22-ceramide Cer 22:0 (in ng/ml) and sphingosine-1-phosphate S1P 18:1 (in ng/ml) in ADHD patients and patients with MD/BP comorbidity (ADHD+) without drug treatment and with drug treatment (w/o = with and without). Numbers of patients receiving specific drugs are indicated. One patients received lisdexamfetamine plus guanfacine. Abbreviations MPH, methylphenidate; LDX, disdexamfetamine; Atomox, Atomoxetine; GXR, guanfacine. **B:** Scatter plots of patients with primary MD/BP without and with treatment with antidepressants and/or anti-neuroleptic agents (NL). Please note that two patients received also Lithium or an anti-epileptic agent. **B:** Scatter plots of the log2 plasma concentrations of Cer 18:0 in ADHD+ (ADHD with comorbidity) and primary MD/BP receiving or not receiving an antidepressant (venlafaxine, buproprion, TCA or SSRI) and w/o anti-psychotic agent (NL or atypNL). Antidepressant and anti-psychotic were combined in 8/12 cases.
